# Long-term neural and physiological phenotyping of a single human

**DOI:** 10.1038/ncomms9885

**Published:** 2015-12-09

**Authors:** Russell A. Poldrack, Timothy O. Laumann, Oluwasanmi Koyejo, Brenda Gregory, Ashleigh Hover, Mei-Yen Chen, Krzysztof J. Gorgolewski, Jeffrey Luci, Sung Jun Joo, Ryan L. Boyd, Scott Hunicke-Smith, Zack Booth Simpson, Thomas Caven, Vanessa Sochat, James M. Shine, Evan Gordon, Abraham Z. Snyder, Babatunde Adeyemo, Steven E. Petersen, David C. Glahn, D. Reese Mckay, Joanne E. Curran, Harald H. H. Göring, Melanie A. Carless, John Blangero, Robert Dougherty, Alexander Leemans, Daniel A. Handwerker, Laurie Frick, Edward M. Marcotte, Jeanette A. Mumford

**Affiliations:** 1 Department of Psychology, University of Texas, Austin, Texas 78712, USA; 2 Department of Neuroscience, University of Texas, Austin, Texas 78712, USA; 3 Imaging Research Center, University of Texas, Austin, Texas 78712, USA; 4 Department of Psychology, Stanford University, Stanford, California 94305, USA; 5 Department of Neurology, Washington University School of Medicine, St Louis, Missouri 63110, USA; 6 Genome Sequencing and Analysis Facility, University of Texas, Austin, Texas 78712, USA; 7 Center for Systems and Synthetic Biology, University of Texas, Austin, Texas 78712, USA; 8 University Medical Center Brackenridge, Austin, Texas 78701, USA; 9 Biomedical Informatics Program, Stanford University, Stanford, California 94305, USA; 10 Olin Neuropsychiatry Research Center, Institute of Living, Hartford, Connecticut 06114, USA; 11 Department of Psychiatry, Yale University School of Medicine, New Haven, Connecticut 06511, USA; 12 South Texas Diabetes and Obesity Institute, University of Texas Rio Grande Valley School of Medicine, Brownsville, Texas 78520, USA; 13 Texas Biomedical Research Institute, San Antonio, Texas 78227, USA; 14 Center for Neurobiological Imaging, Stanford University, Stanford, California 94305, USA; 15 Image Sciences Institute, University Medical Center Utrecht, Utrecht 3584 CX, The Netherlands; 16 National Institute of Mental Health (NIMH), Bethesda, Maryland 20892, USA; 17 Department of Molecular Biosciences, University of Texas, Austin, Texas 78712, USA

## Abstract

Psychiatric disorders are characterized by major fluctuations in psychological function over the course of weeks and months, but the dynamic characteristics of brain function over this timescale in healthy individuals are unknown. Here, as a proof of concept to address this question, we present the MyConnectome project. An intensive phenome-wide assessment of a single human was performed over a period of 18 months, including functional and structural brain connectivity using magnetic resonance imaging, psychological function and physical health, gene expression and metabolomics. A reproducible analysis workflow is provided, along with open access to the data and an online browser for results. We demonstrate dynamic changes in brain connectivity over the timescales of days to months, and relations between brain connectivity, gene expression and metabolites. This resource can serve as a testbed to study the joint dynamics of human brain and metabolic function over time, an approach that is critical for the development of precision medicine strategies for brain disorders.

## Abstract (web summary)

Large-scale, multimodal phenotypic characterisation is a valuable tool to explore brain function. Poldrack *et al*. collect and relate MRI, psychological, physiological, metabolic and gene expression data from a single human over an 18 month period, providing a rich resource for future studies.

## Graphical abstract

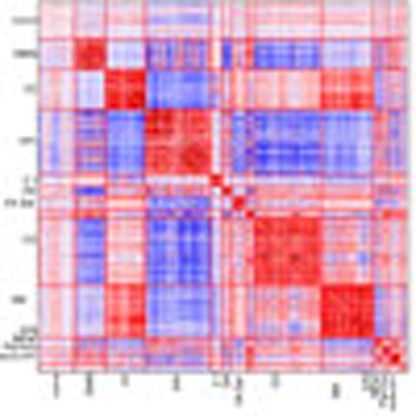

The dynamics of human brain function are increasingly well understood at the short timescale of seconds/minutes (for example, through studies of learning) and the long timescale of years/decades (for example, through studies of development and ageing), but almost nothing is known about how the human brain function varies across the range of days to months. This is a critical gap, because major psychiatric disorders show large fluctuations in brain function over this timescale^1^. However, the kind of dense longitudinal phenotyping that is necessary to understand this question is extremely challenging with healthy human volunteers, who are unlikely to be sufficiently motivated to sustain frequent participation in a study over a long period. For this reason, the participation of motivated experimenters can be uniquely useful for demanding longitudinal studies (cf. ref. 2).

We investigated the long-range dynamics of brain function and their relation to a broad set of psychological and biological variables in a single healthy human (author R.A.P.) over the course of 532 days (along with several follow-up visits), representing one of the most intensive biological characterizations of a single individual ever performed (referred to hereafter as the MyConnectome study). An overview of the study timeline and analysis pipeline is presented in Fig. 1, and a description of the available data types is presented in Table 1. The study was designed to measure the broadest possible range of human phenotypes (the ‘phenome'^3^,^4^) to allow the widespread assessment of relations between psychological, neural and metabolic function.

The results of the present study demonstrate that healthy brain function shows rich dynamics over the course of 18 months, and that these dynamics are paralleled by ongoing fluctuations in psychological and physiological function as observed in behaviour, gene expression and metabolomic measurements. These findings provide a proof of concept for the dynamic longitudinal phenotyping of individuals, which we propose will be crucial to gain a better understanding of the substantial fluctuations in psychological and neural function in individuals with major psychiatric disorders.

Given its size and complexity, this data set serves as an outstanding testbed for open and reproducible scientific practices in a large-scale context. Code, data, quality assurance results and a viewer for detailed results of all statistical analyses reported here are accessible at http://myconnectome.org/wp/data-sharing/ (this will be made public upon publication). A Python package (available at https://github.com/poldrack/myconnectome/) has been created that allows one to automatically download the data and reproduce the statistical analyses reported here on one's own computer; the analyses take less than 5 h on most computers. We have also provided a fully configured virtual machine that will allow users to run the entire set of analyses on their own computer and visualize the results with minimal set-up (https://github.com/poldrack/myconnectome-vm); see Methods for instructions. Some of preprocessing operations for imaging and genomic data required high-performance computing systems due to their scale and are not included in this workflow, but the code is made available. The complete imaging, genomic and behavioural data are made openly available with no restrictions on access/usage or requirements for authorship.

## Results

### Dynamics of functional connectivity

**Figure 1:**
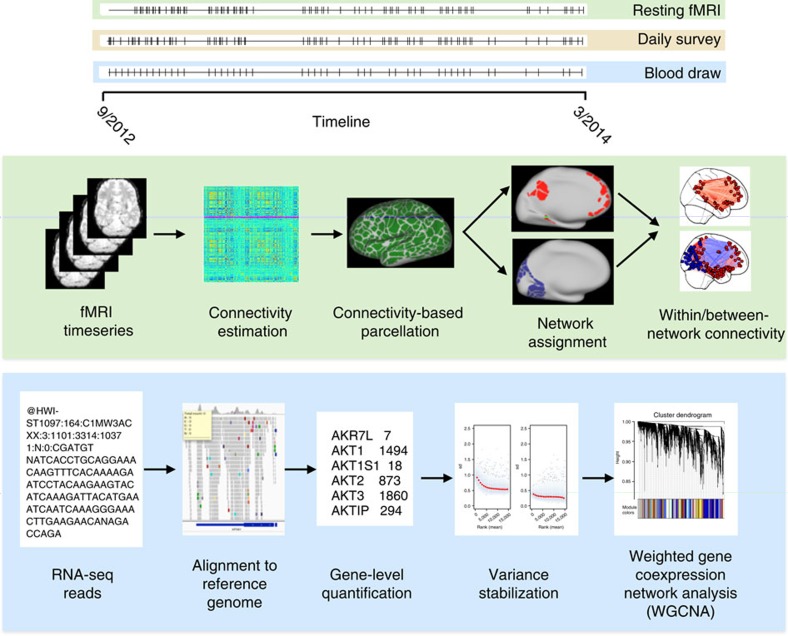
Overview of the MyConnectome study design and analysis pipelines. Top: a timeline of measurements obtained in the study for fMRI, behavioural measurements and blood samples. Each tick represents a single measurement. Middle: an overview of the resting-state fMRI analysis pipeline. Bottom: an overview of the RNA-sequencing pipeline.

Functional, structural and diffusion magnetic resonance imaging (MRI) were used to quantify structural and functional brain connectivity. Of these, the most densely collected measurement was resting-state functional MRI (rsfMRI), which is increasingly recognized as reflecting an underlying organization of brain function^5^,^6^ (Fig. 1). There were 84 usable 10-min rsfMRI runs during the study, which were acquired at consistent times of the day, controlled to minimize effects of time of day. In addition, an extensive set of data was collected in a single follow-up session under both eyes-open and eyes-closed conditions.

**Figure 2:**
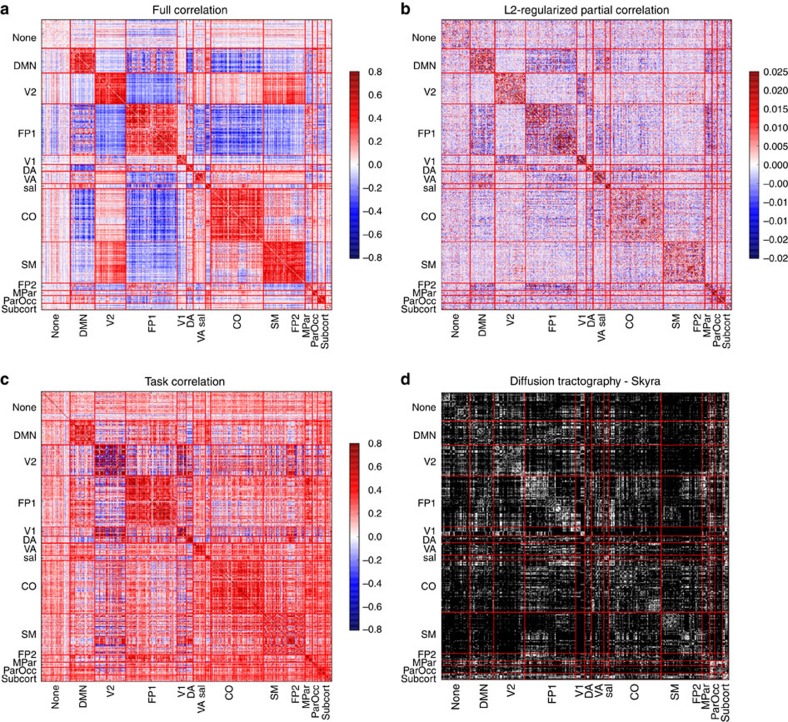
Connectome-wide connectivity across methods. Parcellated connectome matrices for (**a**) full correlation, (**b**) L2-regularized partial correlation, (**c**) meta-analytic task connectivity modelling and (**d**) diffusion tractography (binarized). Networks are sorted by network modules identified from the full correlation connectome. Module labels: none: unassigned, DMN:default mode network, V2: second visual network, FP1: fronto-parietal network, V1: primary visual network, DA: dorsal attention network, VA: ventral attention network, Sal: salience network, CO:cingulo-opercular network, SM:somatomotor network, FP2: secondary fronto-parietal network, MPar:medial parietal network, ParOcc: parieto-occipital network, subcort: subcortical regions.

Detailed analyses of the rsfMRI data are reported in Laumann *et al*.^7^; the additional analyses reported here used the same preprocessing operations. The rsfMRI data were first sampled onto the cortical surface reconstructed from anatomical images, and then parcellated to identify functionally coherent cortical regions^6^. These parcellations were found to be highly reliable across subsets of data^7^. The mean time course from each of these regions was extracted and used to compute a ‘parcellated connectome', reflecting regional functional connectivity across the entire brain (Fig. 2). The community structure of the parcellated connectome was identified using Infomap clustering across sessions to generate a consensus clustering^8^. To further reduce the dimensionality of the data, mean within-network and between-network connectivity was computed for each of these 12 networks, providing summary measures of the functional integration within and between each network.

**Figure 3:**
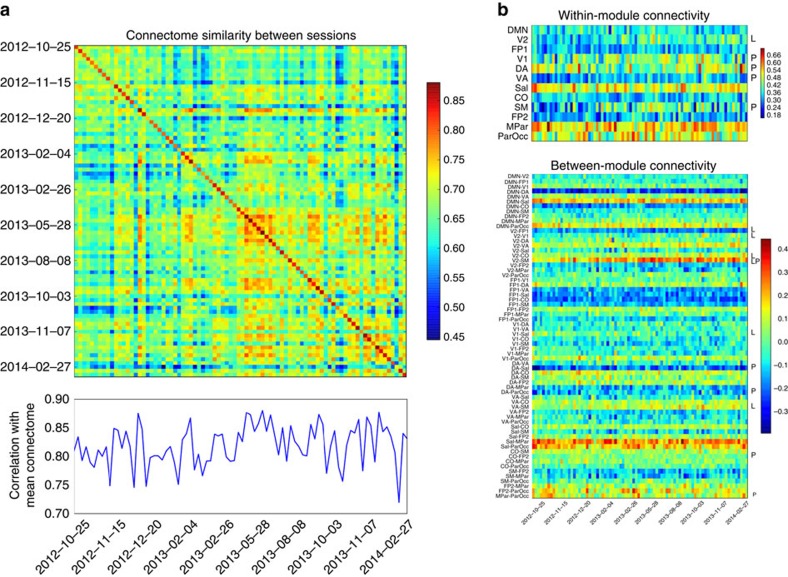
Longitudinal variability in brain connectivity. (**a**) Similarity between connectome-wide connectivity patterns across sessions, computed as the Pearson correlation between the connectivity values across the parcellated connectivity matrix. Values on the diagonal as well as the lower plot represent the similarity between each session and the mean across sessions; off-diagonal elements reflect the similarity between each pair of sessions. (**b**) Time series of connectivity within modules (upper panel) and between modules (lower panel). Notations to the right of each row mark the presence of significant linear (L) and polynomial (P) trends.

Analysis of within-network connectivity over the course of the study (detailed in ref. 7) showed that visual, somatomotor and dorsal attention networks exhibited the greatest degree of variability across sessions. This finding contrasts with results from studies of between-subject variability in connectivity, which have found that primary sensory and motor regions show the least variability across individuals^9^. Time-series modelling of within-network connectivity showed that most networks were largely stable over the 18-month time frame. However, connectivity within primary sensorimotor regions and attention networks showed either linear increases (second visual network) or polynomial trends (V1+, somatomotor, dorsal and ventral attention) over the course of the study (Fig. 3). These results suggest the presence of heretofore undiscovered long-range dynamics in brain connectivity; the source of these dynamics is unclear, as they could reflect effects of repeated measurement such as habituation or sensitization.

We also assessed the long-range temporal dynamics of connectivity across the entire connectome, by comparing each session to the mean across all sessions (Fig. 3). There were no long-range trends in the similarity of each session to the mean, but there were clear groupings of sessions across time, suggesting more complex mesoscale dynamics. Graph–theoretic network metrics were also computed using signed/weighted connectivity (correlation) matrices derived from the parcellated connectome, generating measures of functional segregation (modularity) and functional integration (global efficiency)^10^ for each session. These metrics were stable over time, with neither showing significant linear or polynomial trends.

### Effects of food and caffeine on network structure

Because the subject was fasted and had no caffeine on Tuesdays (owing to the blood draw immediately after the scan), it is possible to compare connectivity between the fed/caffeinated state (on Thursdays) and the fasted/uncaffeinated state from data collected at the same time of the day (hereafter, we use the terms ‘fed' and ‘fasted' to describe these states, which should not be taken to imply that fasting rather than caffeine restriction is the primary causal factor). Laumann *et al*.^7^ reported initial analyses of this effect, showing that connectivity differed within and between somatomotor and dorsal attention networks, with greater connectivity in the fasted state. Using our time-series modelling approach, we found significantly greater within-network connectivity in the fasted state for somatomotor, dorsal attention and primary visual networks. We also found a number of significant effects on between-module connectivity, which largely reflected increased connectivity between those three networks, as well as decreased connectivity between those networks and other networks, on fasted days.

**Figure 4:**
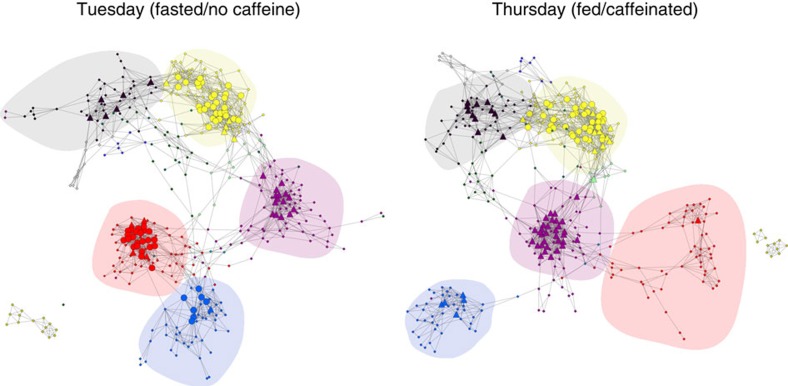
Effects of feeding/caffeine on large-scale network structure. Networks were generated by binarizing the correlation matrices between parcels at a 1% density threshold, separately for Tuesdays (fasted) and Thursdays (fed/caffeinated). Network visualization was performed using yFiles organic layout in Cytoscape. Hubs are shown as larger nodes, with provincial hubs depicted as circles and connector hubs depicted as triangles. Network module membership is coded by node colour; major networks are shaded, including somatomotor (red), second visual (blue), cingulo-opercular (purple), fronto-parietal (yellow) and default mode (black).

To interrogate the network-level effects of this factor, we generated graphs (after binarizing the correlation matrix at a 1% density threshold) and examined the differences between these networks across fed and fasted days (Fig. 4). The networks were largely similar in their modular structure, centred around core modules (default, fronto-parietal and cingulo-opercular networks). However, there was also a substantial degree of reorganization associated with fed/fasted state; in particular, the fasted state was associated with the development of a large meta-module incorporating the somatomotor and second visual networks (blue and red in Fig. 4). To further investigate how modular communication of brain networks was affected by food and caffeine, we computed a measure of the diversity of between-module connections (the participation index)^11^ for each parcel in each session. The participation index was further used to identify the hub structure of the system, classifying hub nodes as either provincial hubs (which are strongly connected to other nodes within their module) and connector hubs (which are strongly connected to nodes in other modules)^12^. The hub structure of these networks showed striking differences (Fig. 4). Nodes in the somatomotor and second dorsal attention networks (which were not hubs in the fed state) and nodes in the second visual network (which were connector hubs in the fed state) transitioned to being provincial hubs during the fasted state. Conversely, there was an increase in the prevalence of connector hubs in the fed state for the default mode and cingulo-opercular networks. These results highlight the importance of physiological states such as feeding and caffeination when measuring the structure of complex brain networks.

### Task activation and functional connectivity

Across sessions, five different task activation paradigms were performed a varying number of times: an *n*-back task with faces, scenes and Chinese characters (15 sessions), a dot-motion stop signal task (eight sessions), an object localizer task with multiple-object classes (eight sessions), a spatial working-memory localizer (four sessions) and a language localizer (five sessions). In addition, we performed a single session of retinotopic visual mapping. Laumann *et al*.^7^ presented evidence for systematic overlap between task activation regions and resting-state parcels, highlighting the utility of rsfMRI for identification of functional architecture (cf. ref. 5).

We assessed the relation between task-based connectivity and resting connectivity using a meta-analytic connectivity-modelling approach^13^; mean values for each parcel were extracted from statistical maps for each of the 237 statistical contrasts (across all sessions and tasks, except for retinotopy), and these were then used to generate a connectivity matrix reflecting the correlation of parcel activation across contrasts. Connectome-wide connectivity values were moderately correlated between task and rest (*r*=0.45). Visualization of the task connectome (Figs 2 and 6) showed that the strongest connections were found in the occipital and parietal cortices, which likely reflects the fact that all of the tasks involved visual stimulation.

### Structural and functional connectivity

**Figure 5:**
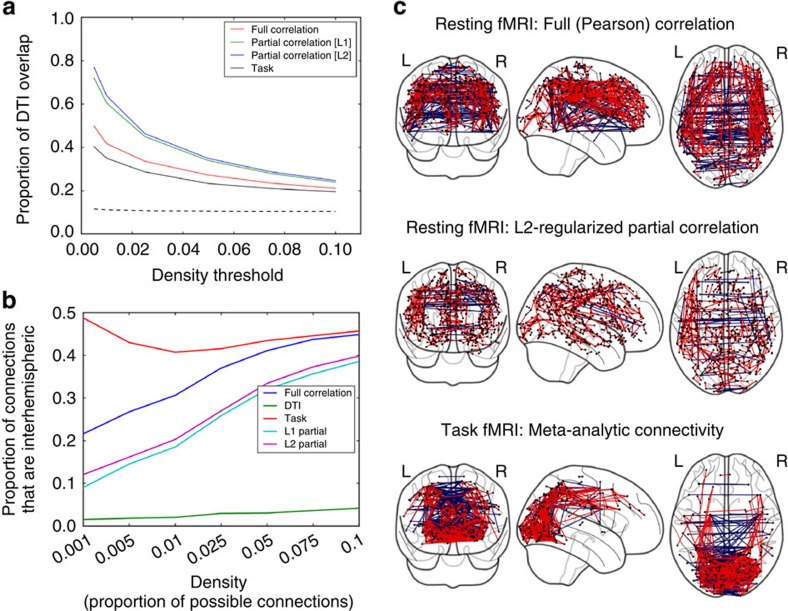
Comparison of fMRI and diffusion connectivity measures. (**a**) Functional connectomes were thresholded at varying densities, and the resulting connections were assessed to identify the proportion of those connections that had non-zero structural connectivity identified using probabilistic diffusion tractography (thresholded at 10% density). The dashed line represents the proportion expected by chance, based on randomization of the structural connections. (**b**) The proportion of connections surviving at each given density that were interhemispheric, at a range of densities, for each measure. (**c**) Functional connectomes were thresholded at 0.25% density and presented in three-dimensional stereotactic space. Red connections had non-zero tractography connections, whereas blue connections had zero tractography connections across 500,000 samples.

Structural connectivity was assessed using diffusion-weighted imaging, which was performed in 15 sessions, each of which included two acquisitions with 30 diffusion directions at two *b*-values (1,000 and 2,000 s mm^*−*2^). An additional follow-up session was performed using high angular resolution diffusion imaging (HARDI: 75 directions at *b*=1,500 and *b*=3,000 s mm^−2^) to allow more precise modelling of diffusion orientation distribution functions. Examination of diffusion orientation maps identified an idiosyncratic structural feature, wherein a small region of approximately anterior–posterior-oriented fibre pathways could be observed within the otherwise left–right-oriented trajectories of the corpus callosum (Fig. 5). This anomalous finding suggests that the data set could be particularly useful for testing methods for the modelling of complex interdigitated white matter structures.

**Figure 6:**
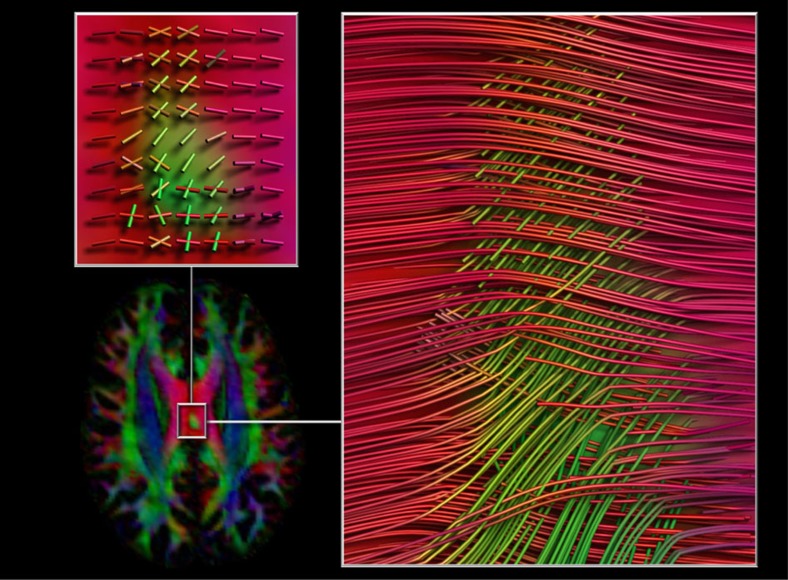
Diffusion tractography results. Diffusion-weighted imaging identified an anomalous feature with the subject's corpus callosum. Glyphs (left top) reflect the underlying dominant-fibre orientation peaks, and tractography image on the right (inferior view) highlights the region with crossing fibres.

Tractography was computed on diffusion data combined across 14 diffusion acquisitions, using the resting-state parcels as seed regions to obtain parcellated structural connectomes analogous to those computed for rsfMRI. These connectomes were compared to assess the relation between structural and functional connectivity; after thresholding at a range of network densities, we assessed the proportion of edges present in the functional connectivity matrix that also had non-zero structural connectivity (diffusion data were thresholded at 10% density to remove weak connections). This proportion decreased as the density increased, showing that the strongest functional connections were also the most likely to be associated with a structural connection, though the relationship was only moderately strong for full (Pearson) correlation (Fig. 6a). To further assess the relation between structural connectivity and functional connectivity, we compared the structural connectome to a partial correlation-based functional connectome, under the assumption that partial correlation should remove the effects of indirect functional connections^14^. Regularized partial correlation measures showed substantially a better match to the structural connectome compared with full correlation, with L2 regularization performing slightly better than L1 regularization. Very similar results were obtained using the HARDI data. The difference between full and partial correlation was primarily driven by the prevalence of short-range connections in the partial correlation map (mean distance=17.6 mm) versus the full correlation map (mean distance=43.4 mm) (Fig. 6c). In particular, diffusion tractography failed to identify interhemispheric connections; a comparison of the proportion of interhemispheric connections at varying connectome densities showed much lower prevalence of interhemispheric connections for diffusion compared with all other measures (Fig. 6b,c).

It is important to recognize that there are fundamental limits on the ability of diffusion tractography to accurately estimate anatomical connectivity^15^,^16^, which may contribute to the considerable number of partial correlations with no measured structural connection according to diffusion. Further, the promise of partial correlations ultimately requires that they do not violate known anatomical connectivity (for example, a strong partial correlation between regions for which it is known there are no direct anatomical connections), which has not been exhaustively tested here. However, the present results represent early evidence that there is a systematic relationship between structural connectivity as measured using diffusion imaging and functional connectivity measured using partial correlation at a connectome-wide level. These observations highlight the utility of this highly sampled data set to assess fundamental questions about the relations between structural and functional connectivity.

### Dynamics of gene expression

Previous work has shown that the assessment of ongoing fluctuations in physiology can provide insights into the dynamics of human health^2^,^17^, and substantial evidence suggests that brain function may be directly related to these physiological states. For example, manipulations of the peripheral immune system can directly modulate social behaviour^18^, and inflammatory cytokines have been implicated in mood disorders^19^. Conversely, cognitive manipulations can modulate physiological responses to foods^20^, and psychological stress directly affects immune function^21^.

To assess physiological variability and its relation to changes in neural and psychological function, we collected whole blood on 48 occasions immediately following an MRI scan. Transcription profiling was performed on RNA extracted from peripheral blood mononuclear cells (PBMCs) to assess gene expression across the whole genome. RNA libraries were prepared according to ref. 22 and RNA sequencing was performed using an Illumina HiSeq 2500, obtaining a mean of 33.58 million reads per session (range 14.9–54.0 million). After alignment to the hg19 reference genome, transcripts were mapped to genes and per-gene read counts were obtained for 23,715 genes. Read counts were normalized for library size, and a variance-stabilizing transform was applied for subsequent analysis; after filtering for expression levels (mean>4 reads per session) and removing small RNAs, 13,072 genes remained in the analysis set.

To further assess the validity of the gene expression data, we compared them with a similar publicly available RNA-seq data set obtained from PBMCs by Snyder *et al*. (http://snyderome.stanford.edu/)^2^. The Snyderome data were processed using the same analysis pipeline, resulting in 15,579 genes passing expression thresholds, of which 13,030 overlapped with the genes in the filtered MyConnectome set; one session in the Snyderome data set (Unk03) was excluded due to extreme expression values. The Spearman correlation for mean expression in overlapping genes across the two data sets was 0.81, demonstrating good concordance in overall expression levels between the data sets despite substantial differences in sample preparation and health status (the participant in the Snyderome study suffered from type-II diabetes during part of the study).

Transcriptome data were clustered using weighted gene coexpression network analysis (WGCNA), a tailored method for identification of highly coexpressed gene sets^23^. The resulting clusters were then functionally annotated to identify enriched biological pathways and functions using DAVID^24^, and the first principal component from each cluster was extracted for further analyses. The application of WGCNA to the RNA-sequencing data identified 63 coexpression modules; of these, 32 were significantly enriched for either specific biological pathways or gene ontology terms (Supplementary Table 1). The largest number of modules were associated with pathways involved in regulation of gene expression (nine modules), immune function (seven modules) and metabolism (seven modules). Additional modules were involved in molecular functions (three modules), signalling pathways (two modules each), cell cycle, development, DNA repair and protein localization (one module each). Overall expression in these modules was relatively stable over time, though several modules showed significant long-range dynamic changes. Significant linear trends were observed in a number of unenriched modules along with modules related to transcription (ME58) and protein localization (ME54), and five unenriched modules showed significant polynomial trends.

We tested the *a priori* hypotheses (recorded before data analysis) that psoriasis severity and mood would be associated with expression in immune/inflammatory pathways. Psoriasis severity had a significant negative association with T-cell receptor-related expression, consistent with the central role of T cells in the disorder. A strong association was also observed with expression related to transforming growth factor-b receptors, consistent with a previous report of association between plasma transforming growth factor-b concentrations and psoriasis severity^25^; an additional weaker association with interleukins was also observed. Surprisingly, no significant associations were observed between mood measures and immune system gene expression.

**Figure 7:**
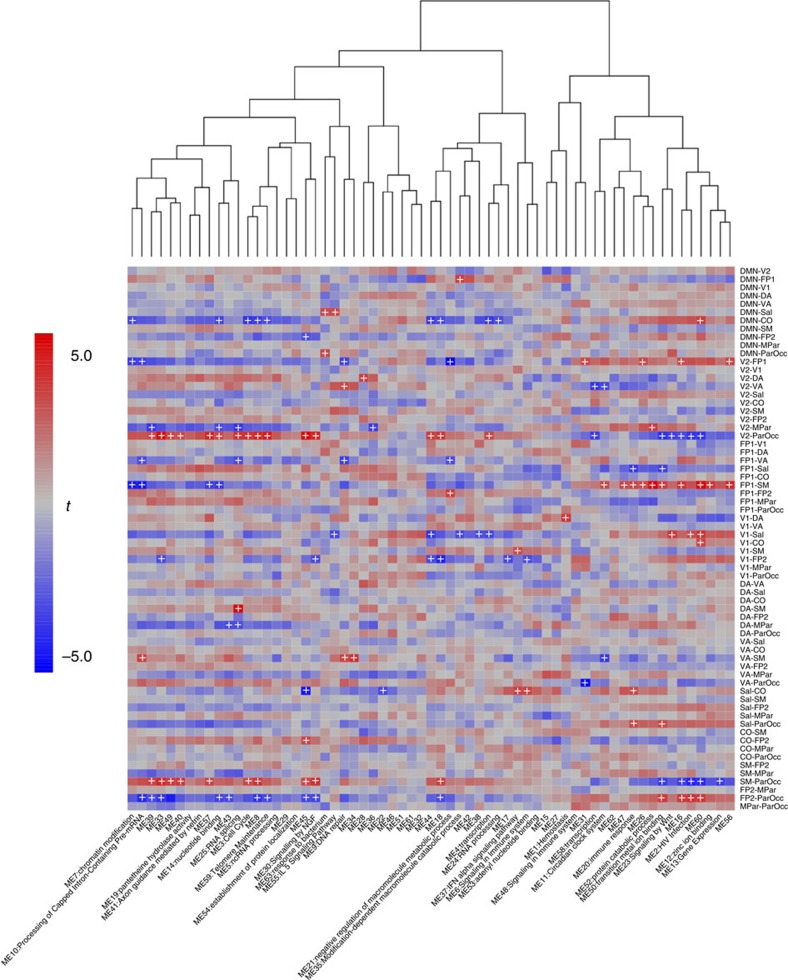
Relations between gene expression and resting-state connectivity. Image shows a heatmap for associations between between-module connectivity in resting-state networks (rows) and gene expression in WGCNA modules (columns). The colour scale reflects the *t*-statistic for association between each pair of variables. Plus signs indicate those sets that are significant at *q*<0.1.

There is a substantial literature relating brain function to genetic variation between individuals^26^ as well as work suggesting that gene expression in peripheral blood may be associated with psychiatric disorders^27^, but no work to date has directly examined relations between peripheral gene expression and brain function. An analysis of associations between gene expression and brain functional connectivity identified a number of significant relationships; there were only a small number of associations with within-network connectivity (eight associations at *q*<0.1), but a number of strong relations with between-network connectivity (Fig. 7). We are cautious not to interpret the observed relations as directly reflecting variability in brain gene expression; rather, we view them as a window into the global metabolic state of the body, which clearly has important connections to brain function (which may occur through either similar or distinct genomic mechanisms).

The gene expression data collected here may also serve as a useful resource for genetic analyses in other studies by providing a heavily sampled longitudinal data set for highly reliable gene network identification. To assess this, we performed an analysis of gene expression (obtained using microarrays) for a large family study, the Genetics of Brain Structure and Function (GOBS) study^28^,^29^. Using the expression data from the study, we performed WGCNA after regressing out principal components of overall expression and single-nucleotide polymorphisms (to reduce population stratification effects). In parallel, we used the gene modules identified using WGCNA on the RNA-sequencing data from the MyConnectome data set to extract module eigengenes from the GOBS expression data (after nuisance regression). Univariate heritability analysis showed that the expression eigengenes extracted using the MyConnectome gene modules had greater heritability (61/63 significantly heritable, maximum *h*_2_=0.76) than those extracted using the gene modules obtained from the GOBS data set (46/55 significantly heritable, maximum *h*_2_=0.49), even though they were identified using a different gene expression method (RNA-seq versus microarray) on an individual from a different ethnic background (Caucasian versus Mexican-American).

### Metabolomic dynamics

The analysis of metabolites in blood provides a complementary view of biological dynamics. Variability in the concentration of small-molecule metabolites (such as fatty acids, sugars and amino acids) was assessed using metabolomic profiling of blood serum via gas chromatography time-of-flight spectrometry. This analysis provided concentrations for 258 compounds, of which 106 were known metabolites. Individual metabolites were clustered using affinity propagation^30^, a successful general-purpose clustering algorithm that identifies clusters around specific exemplars using message passing, adaptively identifying the number of clusters. The resulting clusters were then functionally annotated to identify enriched biological pathways, using Impala^31^, and the first principal component from each cluster was extracted for further analyses. Of the 15 clusters identified, eight showed significant enrichment for biological pathways (at false discovery rate (FDR) *q*<0.1) (Supplementary Table 2). These clusters were primarily centred around pathways involved in glycerophospholipid biosynthesis (two modules), glucose homeostasis and transport (three modules) and fatty acid and triacylglycerol processing (three modules). Linear trends were observed for 7 of the 15 clusters, and polynomial trends for two clusters.

Metabolomic variables were strongly related to food intake. Several significant associations were observed for the metabolomic clusters, and a large number of significant associations were observed for individual metabolites. Some of these are biologically plausible, given the subject's consistent low-carbohydrate diet, such as the strong association of bacon intake with concentration of ketone bodies (acetoacetate and 3-hydroxybutyric acid, along with cluster 15, which was non-significantly enriched for synthesis and degradation of ketone bodies).

### Phenome-wide analysis

This data set provides a unique platform to assess relations across a wide range of different biological and psychological variables. We performed a phenome-wide analysis, assessing bivariate time-series relations across the entire data set using a time-series linear regression approach with automated model order selection^32^. Table 2 provides a list of the strongest associations identified across the entire data set, all highly significant (Bonferroni-corrected *P*<0.05). These findings are necessarily exploratory, given the nature of the present study, but they motivate a set of potential hypotheses that could be prioritized in subsequent follow-up studies. In particular, strong relations were seen between connectivity within and between several resting-state networks (including somatomotor, fronto-parietal, visual and default mode) and gene expression in a number of modules as well as concentrations of three amino acids (glutamic acid, beta-alanine and aninomalonate). These findings suggest that further investigation of relations between brain connectivity and metabolism in a larger sample could provide novel insights into the biological factors that modulate brain connectivity.

**Figure 8:**
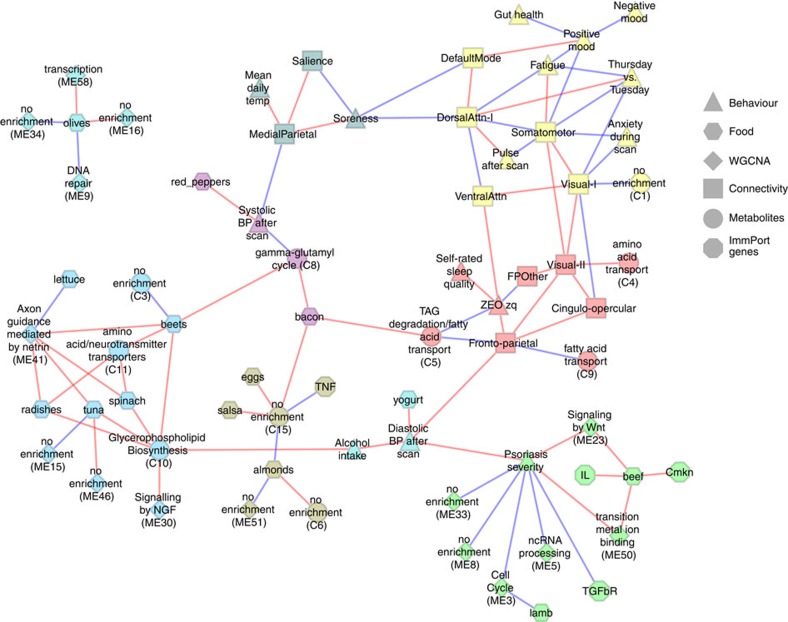
Phenome-wide network analysis. A ‘phenome-wide network' was generated by treating each significant association (FDR *q*<0.05) as an edge in a network. The node shape denotes the variable class, node colour denotes network modules as determined using Infomap clustering and the edge colour represents a sign of association (red: positive, blue:negative).

To visualize the relations across the entire set of variables, we further generated a ‘phenome-wide network' (Fig. 8), which allows a clearer appreciation of the complex web of relations between the different variable classes. The modular structure of the network was identified using Infomap clustering, which revealed a highly modular network (modularity coefficient=0.70), with modules corresponding to sets of strongly associated variables mixed across classes. This visualization highlights the utility of phenome-wide data sets to draw insights into complex relations across different types of variables. For example, one module (yellow) included variables related to mood, connectivity within a number of brain networks, and fatigue and fed/fasted state. Another module (red) included metabolomic modules related to triglyceride degradation and and fatty acid transport, connectivity within fronto-parietal, cingulo-opercular and secondary visual networks, and variables related to sleep quality. This approach provides a novel means to characterize relationships over time across large sets of disparate variables.

## Discussion

Cognitive neuroscience studies have largely assumed that the measurement of brain function at a single time point is representative of the individual more generally. Using the most intensive longitudinal phenome-wide analysis approach to date, we have demonstrated the presence of rich temporal dynamics in brain function related to both psychological and biological variability, suggesting that the purview of studies of human brain function can usefully be widened to encompass the study of temporal variability within individuals. The findings demonstrate a number of potential relationships between functional brain connectivity as measured using rsfMRI, self-report measures of psychological function and physical health, gene expression in PBMCs and serum metabolites.

Large discovery science analyses such as those reported here are primarily useful for the generation of new hypotheses. The results have generated a number of novel associations to be investigated in future studies, as outlined in Table 2. It is crucial to note that the analyses reported here reflect only a first pass at this massive data set, examining a miniscule portion of the space of possible analyses. Additional detailed analyses of the rsfMRI data are reported in (ref. 7), and the open sharing of the data will allow other researchers to examine specific aspects of the data set in greater depth (such as more detailed examination of specific brain systems or gene networks). The data described here provide a basis for power analyses for subsequent longitudinal studies, which will be critical to test the hypotheses generated by this study; for example, 80% power for replication of the observed significant associations between connectivity and gene expression would require anywhere from 24 to 51 sessions, depending on the size of the observed effect (absolute correlations ranging from 0.54 to 0.38, respectively).

The use of a self-experimentation approach raises questions regarding the potential influence of subject expectations on the behavioural and neural measures, as well as biases in the analysis and interpretation of the data. Although steps were taken to minimize this effect (such as avoiding data analysis for the first 6 months of the study), it is impossible to discount these concerns completely. It is, however, more difficult to discern how subject expectations could have driven the observed relations between gene expression, metabolomics and highly derived brain-imaging measures. On the other hand, given the marked time and effort commitment required for completion, it is likely that self-experimentation by a motivated researcher was the only means by which this proof-of-concept study could have been completed.

This study provides justification for larger efforts to longitudinally characterize psychological and brain function with unbiased samples, particularly with regard to psychiatric disorders in which there is greatly increased variability in mental function over the weeks-to-months timescale, for example, see (refs 1, 33). The foregoing results suggest that an intensive phenome-wide analysis approach has the potential to uncover new aspects of brain function and its relation to metabolism that could provide important insights into these disorders.

## Methods

Some of the data described in this methods section were not included in the present paper (primarily due to a lack of sufficient time points for longitudinal analysis), but their descriptions are included for the sake of complete description of the shared data sets.

### Participant

The participant (author R.A.P.) is a right-handed Caucasian male, aged 45 years at the onset of the study. He suffers from plaque psoriasis but is otherwise generally healthy. The participant has a history of anxiety disorder, but no other neuropsychiatric disorders. Before initiation of the pilot period, the participant received a physical examination with a full blood workup (comprehensive metabolic panel (CMP), complete blood count (CBC) thyroid stimulating hormone (TSH), erythrocyte sedimentation rate (ESR) and lipid panel) along with tests for HIV and hepatitis B and C. No significant findings were observed in these tests.

### Ethical review

The present study was submitted to the University of Texas Office of Research Support, which determined that it did not meet the requirements for human subjects research as defined by the Common Rule (45 CFR 46) or FDA Regulations (21 CFR 50 and 56), and thus that Institutional Review Board (IRB) approval was not necessary. Subsequent data collection at Stanford University was performed under a similar determination, while data collection at Washington University was collected under an approved IRB protocol.

### Study phases

A pilot period began on 25 September 2012. During the pilot phase, the initial protocol was tested for several weeks. Upon examination of the data and further discussion with a number of other researchers, several changes were made to the MRI acquisition during the week of 15 October 2012. The production phase began on 22 October 2012 (with scan session 13) and ended on 11 March 2014, with an extended hiatus from 6 March 2013 to 30 April 2013 (further discussed in the Audiometry section below). Additional data were collected on two later occasions; an extensive rsfMRI data set was collected at Washington University on 3 April 2015, and a diffusion MRI data set was collected at Stanford University on 15 May 2015.

### Sample size

The initial sample target was to collect data for one calendar year, yielding roughly 100 samples. However, due to travel and the extended hiatus mentioned above, scanning was extended to obtain sufficient samples. Once the final target of 100 scanning sessions was reached, the final revised stopping criterion was to obtain 48 blood draws (since the RNA-sequencing was performed in batches of six samples), yielding a total of 104 scanning sessions. No time-series statistical analyses were performed before the determination of this criterion.

### Subject blinding

To prevent knowledge of the results from affecting the subject, the subject was initially blinded to the results of any analyses of the repeated tests. He had access to the clinical blood work results, and also examined individual MRI scans for quality control purposes, but was not exposed to any analysis of temporal changes during the initial period of the study. After the hiatus in March 2013, this blinding was discontinued and the subject analysed the first set of data collected to that point. Initial *a priori* hypotheses were recorded before these analyses.

### Imaging procedures

MRI was performed in a fixed schedule, subject to availability of the participant. Scans were performed at fixed times of the day; Mondays at 1700 hours, and Tuesdays and Thursdays were performed at 0730 hours. After the hiatus in March 2013, the Monday sessions were eliminated. Imaging was performed on a Siemens Skyra 3 T MRI scanner using a 32-channel head coil. Parameters described below are for the production phase. The imaging protocols included the following:

### Anatomical MRI

*T*_1_- and *T*_2_-weighted anatomical images were acquired using a protocol patterned after the Human Connectome Project^34^. These data were collected for 15 Monday afternoon sessions (3 during the pilot phase, 10 during the production phase through 30 April 2013, and a 1-year follow-up collected on 4 November 2013 (sub090)). *T*_1_-weighted data were collected using an MP-RAGE sequence (sagittal, 256 slices, 0.8 mm isotropic resolution, echo time (TE)=2.14 ms, repetition time (TR)=2,400 ms, inversion time (TI)=1,000 ms, flip angle=8 degrees, GRAPPA factor=2, 7 min 40 s scan time). *T*_2_- weighted data were collected using a T2-SPACE sequence (sagittal, 256 slices, 0.8 mm isotropic resolution, TE=565 ms, TR=3,200 ms, GRAPPA factor=2, 8 min 24 s scan time).

### Diffusion-weighted imaging

Diffusion-weighted imaging data were collected for 19 Tuesday morning sessions (15 during the production phase) through 30 April 2013. In each session, two diffusion-weighted imaging scans were acquired using a multi-band^35^ Stejskal–Tanner echo-planar imaging (EPI) sequence, with opposite (*L*>*R* and *R*>*L*) gradient readout directions. Two shells of 30 directions each were acquired (*b*=1,000 and 2,000 s mm^*−*2^), plus four low-*b* acquisitions interspersed among the 60 diffusion-weighted images (one every 15 frames). Parameters were *b*=1,000/2,000 s mm^*−*2^, 1.74 × 1.74 × 1.7 mm resolution, 72 axial slices, field of view (FOV)=223 mm, 128 × 128 matrix, TR=5,000 ms, TE=108 ms, GRAPPA factor=2, MB factor=3.

An additional session of HARDI data was collected at Stanford University on 15 May 2015. Data were acquired using 3 × slice acceleration with a blipped-CAIPI shift of FOV/3 and minimum TE (81 ms) using a 5/8 partial-k readout with homodyne reconstruction. Scanner calibrations for RF transmit power can interact with nonuniform RF power across the head (due to B1 inhomogeneity), resulting in excessive RF power and thus over-flipping in the centre of the head. To alleviate this problem, and to keep the peak B1 amplitude under the hardware limit, the prescribed excitation and refocusing flip angles were set to 77 deg and 160 deg, respectively^36^. Seventy-five diffusion-weighted directions were collected at two *b*-values (1,500 and 3,000* *s mm^*−*2^), plus 10 acquisitions with a nominal *b*-value of 0; each direction was collected twice across runs with opposite (A–P and P–A) gradient readout directions.

### Resting-state fMRI

RSfMRI was performed in 100 scans throughout the data collection period (89 in the production phase), using a multi-band EPI sequence (TR=1.16 ms, TE=30 ms, flip angle=63 degrees (the Ernst angle for grey matter), voxel size=2.4 × 2.4 × 2 mm, distance factor=20%, 68 slices, oriented 30 degrees back from AC/PC, 96 × 96 matrix, 230 mm FOV, MB factor=4, 10:00 scan length). Starting with session 27 (December 12 2012), the number of slices was changed to 64 because of an update to the multi-band sequence that increased the minimum TR beyond 1.16 for 68 slices. Acoustic noise cancellation for the resting-state scan was attempted in each session using the Optoacoustics active noise cancellation system, but in some runs the system failed to cancel the noise successfully (which was noted in the scan database). The first minute of acquisition was discarded before subsequent analyses to exclude fMRI responses evoked by the start of the scan and the noise-cancelling headphones. Five full production-phase sessions were also excluded from analysis due to low signal to noise ratio (SNR) that led to poor registration (identified by visual inspection), resulting in 84 sessions that were included in analysis.

An additional session of rsfMRI data was collected at Washington University on 3 April 2015 to assess cross-site scanner and sequence effects, as well as the effect of eyes open versus eyes closed on resting-state networks. The data were collected on a 3-T TIM TRIO system with a standard (non-multi-band) EPI sequence on a 12-channel coil, with TR=2.5 s and 4-mm isotropic voxels. This data set included ten 10-min runs of eyes-closed resting-state data and ten 10-min runs of eyes-open resting-state data (with fixation crosshair).

### Field mapping

A dual-gradient echo-field map sequence was acquired with the same prescription as the functional images. In addition, spin echo field maps were collected with A–P and P—A phase encoding. Collection of field maps was discontinued as of 30 April 2013, resulting in 38 acquisitions.

### Task fMRI: *n*-back

An *n*-back task was performed using a blocked design, with a factorial combination of memory load (1 versus 2 back) and stimulus type (faces, houses and Chinese characters) across blocks. Twenty percent of items were targets, and 20% were foils. The acquisition sequence was identical to that used for the rsfMRI scan (acquisition time=8 min). Weekly acquisition was performed 15 times across different sessions.

### Task fMRI: motion/stop signal

A motion discrimination task with an embedded stop signal task was performed eight times across different sessions. On each trial, a moving dot stimulus was presented, with coherence of either upward or downward motion varying across trials (levels: 0, 10, 30 and 70% coherence). On 25% of trials, a visual stop signal (change of the fixation cross from white to red) was presented, at a delay controlled by a 1-up/1-down staircase to ensure 50% stopping accuracy^37^. The subject's task was to perform the motion discrimination as quickly as possible, but withhold responses when the stop signal occurred. The MRI acquisition was identical to that used for the rsfMRI scan (acquisition time=7 min 11 s).

### Task fMRI: object localizer

A multiple-object localizer (including both cropped and naturalistic faces, human bodies, human limbs, houses, places, cars, guitars, words and numbers) was performed eight times (twice each across four sessions); this task was kindly provided by K. Grill-Spector from Stanford University. Each stimulus class was presented in 4-s mini-blocks with items presented at 2 Hz (eight items per mini-block). In each run, 12 mini-blocks of each class were presented along 12 interspersed 4-s fixation blocks (acquisition time: 5 min 13 s). Half of the blocks included a single phase-scrambled image; the subject's task was to press a button whenever a phase-scrambled item appeared.

### Task fMRI: language localizer

A verbal working-memory localizer^38^ was performed five times across separate sessions. In each trial, a string of 12 words (either a sentence or a string of non-words) was presented sequentially (400 ms per word), followed by a 1-s probe item; the subject's task was to decide whether the probe item matched any of the words in the preceding string.

### Task fMRI: spatial working-memory localizer

A spatial working-memory localizer^39^ was performed four times across separate sessions. On each trial, a 4 × 2 spatial grid is presented, and locations in that grid are presented sequentially (1,000 ms per location), followed by a forced-choice probe between two grids, one of which contained all of the locations presented in the preceding series. In the easy condition, one location is presented on each presentation, whereas in the hard condition two locations are presented on each presentation. Twelve 32-s experimental blocks were interspersed with 4 16-s fixation blocks (acquisition time=7 min 28 s).

### Task fMRI: retinotopic mapping

Polar angle (with reference to the vertical meridian, with the centre of fovea as the origin) was mapped using a flickering checkerboard wedge (45 degree) that rotated periodically in a counterclockwise direction through the visual field with a cycle duration of 20 s. As the wedge rotates, it creates a wave of activation throughout retinotopically organized visual areas, successively and systematically stimulating portions of each map. In this way, the entire visual field is represented by a time-dependent pattern of activity across space. In each fMRI run, the wedge completed 12 cycles of rotation (240 s total). The acquisition sequence was identical to that used for the rsfMRI scan (acquisition time=4 min).

### Breath-holding fMRI

A breath-holding task^40^ was performed 18 times across different sessions. Visual cues instructed the subject to breathe in and out every 6.96 s (3.48 s inhale, 3.48 s exhale), followed by a 19.72-s breath hold; all events were time-locked to image acquisition. The MRI acquisition was identical to that used for the rsfMRI scan (acquisition time=6 min 8 s).

### Other measurements

A set of questionnaires was administered in the morning and evening and after each scanning session (see Supplementary Note 1 for complete listing of questions). All survey data were collected using a custom survey created with the appsoma.com system, and saved to a CouchDB database server for later analysis. All scores were rectified so that values were consistent with the name of the variable.

Naked weight and estimated body fat were measured upon waking using the FitBit Aria scale. Weight remained within a relatively small range during the study (mean=68.0 kg, range: 65.8–69.9 kg), showing a slow rise over the first 10 months of the study and a decline afterwards.

Sleep was measured on most nights before scanning sessions using ZEO sleep monitor. Data were missing for a number of nights due to the sensor falling off or sensor failure.

Daily weather data (high- and low-temperature and precipitation) were obtained from NOAA (http://cdo.ncdc.noaa.gov/qclcd/QCLCD?prior=N&callsign=ATT).

### Audiometry

During the pilot period, the participant noticed an increase in the level of his pre-existing tinnitus. Because of the potential for noise-induced hearing damage, baseline audiometry was performed at the UT Speech and Hearing Clinic on 16 October 2012 (within 8 h of a scanning session), 17 October 2012 and 19 October 2012 (both roughly 26 h after the previous scanning session). These exams showed moderate hearing loss (40–60 dB HL) in the high-frequency range (at or above 3 kHz), which was consistent across the two sessions. In addition, there was a slight (10–15 dB HL) loss in the lower frequencies, which was restored by 5 dB in the later tests, suggesting a potential short-term effect possibly due to seasonal allergies. Distortion product otoacoustic emission (DPOAE) testing on the first testing day showed consistent results, with impaired function at 3 and 4 kHz. Tympanometry and reflex threshold testing were performed at the first exam, and were normal. These findings are consistent with noise-related hearing loss that was almost certainly pre-existing at the onset of scanning (based on the subject's history of environmental noise exposure and long-standing tinnitus), though the lack of previous audiometry makes this impossible to confirm. Audiometry was repeated regularly to assess potential further hearing damage. On 6 March 2013, audiometry showed increased hearing loss, increasing to 70 dB HL at 6,000 Hz. For this reason, imaging was temporarily stopped. The protocol was re-started on 30 April 2013 with a more limited set of measurements (that is, limited to functional acquisitions for which the active noise cancellation system could be employed). Follow-up testing was performed on 1 May 2013, which showed that the apparent loss was not sustained. Final testing after completion of the study showed no clinically significant changes from the initial tests.

### Omics profiling

Blood was drawn every Tuesday around 0800 hours, following the MRI scan, by a phlebotomist at the UT Student Health Center Laboratory. The subject abstained from food and beverages (other than water) from 2000 hours the previous night until after the blood draw was completed. The standard blood draw included two 10 ml venous blood collection tubes (lavender K+/EDTA), which were transported immediately to the UT Genomic Sequencing and Analysis Facility for processing. In addition, a 5-ml lavender EDTA tube was collected once per month for CBC/differential analysis. On several occasions, additional blood was taken to perform comprehensive metabolic panels. A total of 48 samples were collected.

PBMCs were harvested by Ficoll gradient from whole blood. The PBMC population was then split into two equal fractions: one washed in PBS and frozen for future use and one immediately placed in lysis solution to eliminate nuclease activity for RNA isolation. Plasma was saved for future analysis.

### RNA sequencing

RNA was extracted using the Qiagen RNeasy Mini Kit. RNA integrity number (RIN) was measured using an Agilent Bioanalyzer. RNA libraries were prepared for sequencing according to vendor protocols using NEBNext R Small RNA Library Prep Set for Illumina R (Multiplex Compatible), Cat #E7330L, according to the protocol described by Podnar *et al*.^22^ RNA was fragmented using elevated temperature in carefully controlled buffer conditions to yield average fragment sizes of 200 nucleotides. These fragments were directionally ligated to 5′ and 3′ adaptors so that sequence orientation is preserved throughout sequencing. Reverse transcription and PCR were performed to complete the DNA sequencing libraries, which were then sequenced on the HiSeq 2500.

### Metabolomics

Metabolomic profiling was performed by the West Coast Metabolomics Center at UC Davis^41^. Thirty-microlitre aliquots of serum are extracted by 1 ml of degassed acetonitrile: isopropanol: water (3:3:2, v/v/v) at 20 °C, centrifuged and decanted with subsequent evaporation of the solvent to complete dryness. A clean-up step with acetonitrile/water (1:1) removes membrane lipids and triglycerides. The cleaned extract is aliquoted into two equal portions and the supernatant is dried down again. Internal standards C08-C30 FAMEs are added and the sample is derivatized by methoxyamine hydrochloride in pyridine and subsequently by *N*-methyl-*N*-trimethylsilyltrifluoroacetamide for trimethylsilylation of acidic protons. Data are acquired using the following chromatographic parameters, with more details to be found in ref. 42. Column: Restek Corporation rtx5Sil-MS (30 m length × 0.25 mm internal diameter with a 0.25-μm film made of 95% dimethly/5%diphenylpolysiloxane), mobile phase: helium, column temperature: 50–330 °C, flow rate: 1 ml min^−1^, injection volume: 0.5 μl, injection: 25 splitless time into a multi-baffled glass liner, injection temperature: 50 °C ramped to 250 °C by 12 °C s^−1^, gradient: 50 °C for 1 min, then ramped at 20 °C min^−1^ to 330 °C, held constant for 5 min. A Leco Pegasus IV mass spectrometer is used with unit mass resolution at 17 spectra per second from 80 to 500 Da at −70 eV ionization energy and 1,800 V detector voltage with a 230-°C transfer line and a 250-°C ion source. ChromaTOF (version 2.32) was used for data preprocessing without smoothing, 3 s peak width, baseline subtraction just above the noise level and automatic mass spectral deconvolution and peak detection at signal/noise levels of 5:1 throughout the chromatogram. Apex masses were submitted to the BinBase algorithm using the settings: validity of chromatogram (<10 peaks with intensity >107 counts per s), unbiased retention index marker detection (MS similarity >800, validity of intensity range for high-*m*/*z* marker ions), retention index calculation by 5th-order polynomial regression. Spectra are cut to 5% base peak abundance and matched to database entries from most to least abundant spectra using the following matching filters: retention index window±2,000 units (equivalent to about±2 s retention time), validation of unique ions and apex masses (unique ion must be included in apexing masses and present at >3% of base peak abundance), mass spectrum similarity must-fit criteria dependent on peak purity and signal/noise ratios and a final isomer filter. Peak height values were normalized across samples by dividing by the mean peak height across all metabolites. For the purposes of further analysis, only metabolites with known chemical names were used (106 named metabolites out of 258 total).

### Genomics

Genotyping was performed commercially by 23andMe; it was originally performed on the V2 platform and then updated to the V3 platform, giving a total of 996,000 single-nucleotide polymorphisms. In addition, whole-exome sequencing was performed. At the first and third collection, 200 μl of whole blood were used for DNA isolation. Ten micrograms of genomic DNA were used to create next-generation sequencing libraries for exome sequencing. The standard Illumina DNA fragment library protocol was used to shear, end-repair, dA-tail and ligate sequencing adaptors.

These finished libraries were enriched using the Nimblegen SeqCap kit.

### Data analysis

Analysis code is openly accessible at https://github.com/poldrack/myconnectome and http://www.nil.wustl.edu/labs/petersen/ Resources.html.

A fully reproducible analysis workflow was developed for the present data set using virtual machine provisioning. Using this system, it is possible to replicate all of the reported statistical analyses on one's own computer. The system installs a virtual machine and all necessary software, downloads the necessary data and runs the analyses. To use the virtual machine:

1. Install the necessary software dependencies:
2. Move to the directory where you want to house the project, and then clone the myconnectome vagrant set-up using the following command: git clone https://github.com/poldrack/myconnectome-vm.git
3. cd into the vm directory: cd myconnectome-vm
4. set up the vagrant VM (which may take a significant amount of time) using the command: vagrant up The final step will automatically start the analysis processes, which will take several hours to complete. Using a web browser on your local machine, you can view the analysis status and results at http://192.128.0.20:5000. A precomputed version of the same results can be viewed at http://results.myconnectome.org.

### Time-series analyses

Time-series analyses relating the different types of data (behavioural/imaging/omics) were performed in R. Initial examination of the time-series data showed significant autocorrelation in many of the variables, so an autoregressive modelling approach was employed using the auto.arima function within the forecast package for R^32^, which automatically determines the appropriate autoregressive model order and tests for association between variables. Because of the uneven missingness across variables, each analysis was performed in a bivariate manner, such that correlations between variables were not accounted for in the analysis. Control for multiple tests was achieved in these analyses using the Benjamini–Hochberg FDR correction at *P*<0.1, with additional thresholding for absolute Pearson correlation >0.2 to exclude occasional significant results with small observed correlations.

### Email analysis

Sent emails were obtained for the period of the study, and were preprocessed to extract the body text (excluding signatures and included messages) and to remove common proper names. All texts were scanned for words from the LIWC 2007 dictionary using RIOT Scan word counting software (version 1.8.2)^43^, available from http://riot.ryanb.cc. Three measures were used: positive words, negative words and the categorical dynamic index, which reflects the prevalence of terms reflecting categorical versus dynamic thinking^44^.

### fMRI preprocessing

All fMRI data were preprocessed according to a pipeline developed at Washington University, St Louis^45^. Data were realigned to correct for head motion and normalized to a mode of 1,000 (one multiplicative constant applied to all voxels and all frames). Each session was registered to a single session that had previously been registered to the mean T_1_-weighted structural image and an atlas. The session-to-atlas transform was inverted and applied to the mean field map so that the distortion correction could be applied in each session's space. The undistorted data were then re-registered to the atlas space. The transforms for head motion correction and affine registration to atlas space were combined with the field-map-based distortion correction to resample the data from the original session space to the undistorted 3-mm isotropic atlas space in a single step using FSL's applywarp tool^46^.

Because field maps were not available for all sessions, a mean field map was generated from 34 available field maps (after exclusion of four poor-quality field maps based on visual inspection). Magnitude images were registered to each other, and transforms were resolved (by reconstructing the *n*−1 transforms between all images using the n*(*n*−1)/2 computed transform pairs^47^) and applied to generate a mean magnitude image. The mean magnitude image was registered to an atlas representative template, and transforms from magnitude image to atlas space were computed for each session by combining the session-to-mean and mean-to-atlas transforms. Phase images were then transformed using the composed transforms modified to eliminate intensity scaling (which was relevant only for the magnitude images), and a mean phase image in atlas space was computed.

Artefacts were reduced using frame censoring, regression (excluding censored frames) and spectral filtering^45^. Frames with framewise displacement >0.25 mm were censored, as well as uncensored segments of data lasting fewer than five contiguous volumes (frames kept: 97.1±3.7%). Nuisance regressors included whole brain, white matter and ventricular signals and their derivatives, in addition to 24 movement regressors derived by Volterra expression^48^. Interpolation over censored frames was computed by least-squares spectral estimation, so that continous data could be bandpass filtered (0.009<*f*<0.08 Hz).

### fMRI surface mapping and parcellation

Surface generation and sampling of functional data to anatomical surfaces followed a similar procedure as described in Glasser *et al*.^49^ and Wig *et al*.^50^ First, following volumetric registration, anatomical surfaces were generated from the subject's MP-RAGE image using FreeSurfer's default recon-all processing pipeline (version 5.0). This pipeline included brain extraction, segmentation, generation of white matter and pial surfaces, inflation of the surfaces to a sphere, and surface shape-based spherical registration of the subject's native surface to the fsaverage surface^51^,^52^,^53^,^54^. The fsaverage-registered left and right hemisphere surfaces were brought into register with each other using deformation maps from a landmark-based registration of left and right fsaverage surfaces to a hybrid left–right fsaverage surface (fs LR^55^) and resampled to a resolution of 164,000 vertices (164k fs LR) using Caret tools^56^. Finally, the subject's 164k fs LR surface was downsampled to a 32,492 vertex surface (fs LR 32k), which allowed for analysis in a computationally tractable space while still oversampling the underlying resolution of BOLD data used in subsequent analyses. The various deformations from the native surfaces to the fs LR 32k surface were composed into a single deformation map allowing for one-step resampling. The above procedure results in a surface space that allows for quantitative analysis across subjects as well as between hemispheres. A script for this procedure is available on the Van Essen Lab website (http://brainvis.wustl.edu/wiki/index.php/Caret: Operations/Freesurfer_to_fs_LR).

Surface processing of the BOLD data proceeded through the following steps. First, the BOLD volumes are sampled to the subject's individual native mid-thickness surface (generated as the average of the white and pial surfaces) using the ribbon-constrained sampling procedure available in Connectome Workbench (version 0.7). This procedure samples data from voxels within the grey matter ribbon (that is, between the white and pial surfaces) that lay in a cylinder orthogonal to the local mid-thickness surface weighted by the extent to which the voxel falls within the ribbon; it is designed to minimize partial-volume effects arising from the low sampling resolution of the BOLD data relative to the structural image acquisition^57^. Voxels with a time-series coefficient of variation 0.5 s.d. higher than the mean coefficient of variation of nearby voxels (within a 5-mm sigma Gaussian neighbourhood) are excluded from the volume to surface sampling, as described in ref. 49. This procedure is designed to avoid sampling highly variable voxels to the surface that likely contain large blood vessels that sit outside brain tissue. Once sampled to the native surface, time courses were deformed and resampled from the individual's native surface to the 32k fs LR surface in a single step using the deformation map generated as described above. Finally, the time courses were smoothed along the 32k fs LR surface using a Gaussian smoothing kernel (*s*=2.55).

These surfaces are then combined with volumetric subcortical and cerebellar data into the CIFTI format using Connectome Workbench^49^, creating full brain time courses that exclude non-grey matter tissue. Subcortical (including accumbens, amygdala, caudate, hippocampus, pallidum, putamen and thalamus) and cerebellar voxels were selected based on the Freesurfer segmentation of the individual subject. Volumetric data was smoothed within this mask with a Gaussian kernel (*s*=2.55) before being combined with the surface data.

### Individual subject parcellation

An individual subject parcellation was generated following the procedures described in detail in Gordon *et al*.^6^ and Wig *et al*.^50^, with minor modifications related to processing single-subject as opposed to group average data. In brief, for each hemisphere, whole-brain CIFTI-space correlation maps were computed at every surface vertex from the BOLD time courses concatenated across all sessions. For each vertex, spatial gradients of the similarity of resting-state correlation maps were computed along the cortical surface. Edges in the spatial gradients were identified by the watershed transform^58^ and averaged across all vertices to generate an ‘resting state functional connectivity (RSFC)-boundary map', indicating the frequency with which a given vertex was identified as an edge. To produce discrete parcels, the watershed transform was applied again starting from all local minima. Parcels were merged together if they were considered insufficiently dissimilar based on the edge frequency value (below the 55th percentile) in the RSFC-boundary map. We then eliminated all parcels and portions of parcels in vertices with high boundary-map values (top quartile of values in the boundary map), and parcels containing fewer than 20 cortical vertices (∼40 mm^2^). This procedure generated a 616-region parcellation that forms the basis for many of the subsequent analyses. An additional 14 subcortical regions were obtained from the Freesurfer subcortical parcellation, giving a total of 630 regions for subsequent analysis.

### Module assignment

The modular structure of the parcel-wise graph was derived using the Infomap algorithm^8^, following Power *et al*.^59^ The parcel-wise adjacency matrix was computed by cross-correlating the average time courses from each parcel concatenated across all sessions. Connections were removed if the geodesic distance between parcel centroids was <30 mm. Module assignment was computed at several correlation thresholds (ranging from 1 to 6% edge density, in steps of 1%). Modules with eight or fewer parcels were eliminated from consideration, and those parcels were considered unassigned. A ‘consensus' assignment was derived by collapsing across thresholds, giving each node the assignment it has at the sparsest possible threshold at which it was successfully assigned. Small communities that were only present at a single threshold were removed.

### Resting fMRI: network analyses

For each of the regions identified in the foregoing parcellation schemes, we estimated connectivity matrices using both full (Pearson) correlation and partial correlation; censored time points were excluded from computation of these correlation matrices. L1-regularized partial correlation was estimated using the graphical lasso implemented in the QUIC R package^60^; the regularization parameter was iteratively modified for each session to identify the value at which the network had a density of 7.5%, and mean across sessions was thresholded to obtain the specified level of network density. L2-regularized partial correlation was estimated for each session using the rags2ridges R package^61^ with lambda=0.0001. Graph–theoretic metrics of modularity, global efficiency and participation index were computed using the Brain Connectivity Toolbox http://www.brain-connectivity-toolbox.net/ using unthresholded (that is, signed and weighted) correlation matrices.

### Diffusion-weighted imaging processing

Diffusion data were processed using the FSL Diffusion Toolbox. Using the pairs of images with opposite phase encoding, the susceptibility-induced off-resonance field was estimated using a method similar to that described in Andersson^62^ as implemented in FSL's TOPUP tool, and the two images were combined into a single corrected one. Simultaneous correction of eddy-current effects and head motion was performed using the FSL EDDY tool. Diffusion parameters were estimated at each voxel using BEDPOSTX with a two-fibre model. Probabilistic tractography was performed using prob trackx2. The cortical and subcortical parcels were spatially transformed into the space of the diffusion data by registering the low-*b* image to the main anatomical image and then inverting the warp. Tracking was performed using each parcel as a seed voxel, with all other parcels specified as termination masks, white matter specified as a waypoint mask and the cerebrospinal fluid (CSF) mask specified as a rejection mask; the prob trackx2 distance correction was used. A total of 500,000 samples was performed from each seed. Tract counts were summarized and used to generate binarized adjacency matrices with a given tract density, for comparison with resting-state connectomes.

### Whole-exome sequencing analysis

Alignment to the hg19 reference genome was performed using BWA-MEM (version 0.7.4)^63^. PCR duplicates were removed using the samtools dedup tool. The GATK IndelRealigner module was used to correct misalignments due to indels, and base quality scores were recalibrated using the GATK BaseRecalibrator tool. Variant calling was performed using the GATK Unified Genotyper and VariantRecalibrator tools.

### RNA-seq analysis

Initial quality assurance was performed using FastQC (http://www.bioinformatics.babraham.ac.uk/projects/fastqc/). Examination of RIN values showed five sessions with RIN below the commonly used threshold of RIN 6. However, further examination of expression profiles using clustering did not identify these sessions as outliers, and they were thus included in the analysis to maximize sample size, with RIN values included as a nuisance covariate.

Paired-end reads were mapped to the hg19 reference genome using bowtie2 (version 2.2.2) via tophat (version 2.0.11)^64^. Per-gene read counts were obtained using htseq-count (HTSeq package, version 0.5.4p5) (http://www-huber.embl.de/users/anders/HTSeq/doc/index.html), resulting in counts for 23,715 genes.

For subsequent analyses using general statistical analysis tools (which are not adapted to analysis of overdispersed count-valued data such as RNA-seq reads), we generated variance-stabilized versions of the read counts. Read counts were first normalized for library size using the estimateSizeFactors function (DeSeq package, version 1.14.0)^65^, and genes were then filtered for a mean read count across sessions of at least 4 and no more than 10,000, as well as filtering out small RNAs (using the VEGA database^66^), resulting in 13,847 genes passing filtering (removing 4,133 small RNAs, 5,508 below threshold and 221 above threshold). The relationship between mean and dispersion of read counts was estimated using estimateDispersions (DeSeq) and a variance-stabilizing transform was applied using varianceStabilizingTransformation (DeSeq). Subsequent visual examination of clustering across subjects and genes showed no visible evidence of outliers.

### Gene network analysis

Gene networks were identified from the RNA-seq data using WGCNA^23^, which was applied to the variance-normalized count data, using the WGCNA R package^67^ (version 1.47). Before performing WGCNA, each gene was regressed against RIN values for each session along with the top three principal components estimated across all genes (to remove global effects due to technical variation across sessions), and the residuals were used for the WGCNA analysis. The power for soft thresholding was chosen as 8 based on the scale-free criterion. Correlations were estimated using a robust bicorrelation mid-weight estimator. The resulting networks were further functionally characterized using DAVID^24^. The set of genes associated with each module was submitted to DAVID for functional annotation. The default background set (comprising all human genes) was used. Two separate annotation analyses were used. One focused on curated biological pathways, using the following pathway databases: Reactome^68^, Panther^69^ and BioCarta (http://www.biocarta.com/). A second analysis used Gene Ontology biological processes and molecular functions; while this database includes a larger number of annotated gene associations, most of these are not manually curated or experimentally verified, and thus are more likely to reflect false positives^70^.

### Phenome-wide network analyses

All of the different data types were combined using a phenome-wide network analysis approach. For this approach, the associations between each pair of variables (for example, all behavioural phenotypes versus all metabolites) were computed using the automated ARIMA model selection approach and thresholded at FDR *q*<0.05. To make the graph more interpretable, within-type connections were excluded for gene modules and metabolites; in addition, graph–theoretic functional connectivity measures were excluded because they are derivative of the other connectivity measures included in the analysis. Significant associations (either positive or negative) between variables were treated as edges in the graph. Clustering was performed using Infomap implemented in igraph (version 0.7.1), and visualization was performed using the Cytoscape software package^71^ (version 3.2.1).

### GOBS data set

The GOBS study sample^28^ consists of Mexican-American individuals from large extended pedigrees sampled randomly from the San Antonio community. The sample analysed for the present study (which included all subjects for whom fMRI and gene expression data had passed quality assurance) included 591 invididuals (236 male, mean age 43 years, range 18–85). All experiments were performed with IRB approval from the University of Texas Health Science Center at San Antonio (UTHSCSA). All participants provided written informed consent on forms approved by the Institutional Review Boards at the UTHSCSA and Yale University.

Transcriptomic methods for the GOBS study were similar to those previously described in detail by Sprooten *et al*.^29^ Briefly, peripheral blood samples were obtained in the morning after an overnight fast during the MRI clinic visit of study participants, and lymphocytes were isolated from the fresh samples and subsequently frozen and stored. Genome-wide transcriptional profiles were generated using the Illumina HumanHT-12 v3.0 Expression BeadChip, which contains more than 47,000 unique probes in total, hybridization to which is assessed at 30 different beads on average.

Individual probes were mapped to genes, taking the probe with the highest mean expression level across subjects for each gene^72^. These gene-level expression measures were then combined using the gene clusters identified from the MyConnectome data set, as well as clusters identified by performing WGCNA directly on the GOBS data. In each case, the first principal component was computed across genes to obtain a cluster eigengene for each cluster and all subjects, after removing the top five principal components computed across all transcripts as well as the top 10 genotypic principal components to reduce effects of admixture. Heritability of expression was assessed using the SOLAR software package^73^ (http://solar.txbiomedgenetics.org/).

## Additional information

**Accession codes:** Raw MRI data have been deposited at OpenfMRI.org (accession number ds031). RNA-seq summary data have been deposited at Gene Expression Omnibus (accession number GSE58122), and raw RNA-seq reads have been deposited in the Sequence Read Archive (accession number SRP042596).

**How to cite this article:** Poldrack, R. A. *et al*. Long-term neural and physiological phenotyping of a single human. *Nat. Commun.* 6:8885 doi: 10.1038/ncomms9885 (2015).

## Supplementary Material

Supplementary Information Supplementary Tables 1-2 and Supplementary Note 1

## Figures and Tables

**Table 1:** Description of raw data available for the MyConnectome project.

Scan type	Total	Usable
*T*_1_-weighted anatomy	21	10
*T*_2_-weighted anatomy	16	11
Diffusion-weighted (pair of scans)	19	15
Resting fMRI	100	84
Breath-holding fMRI	18	18*
Task fMRI: *n*-back	15	15
Task fMRI: dot-motion stop signal	9	8
Task fMRI: object localizer	8	8
Task fMRI: spatial working-memory localizer	4	4
Task fMRI: language localizer	5	5
Retinotopic mapping	1	1
Gradient echo field map	38	34
RNA-sequencing 74	48	48
Metabolomics	48	48

fMRI, functional magnetic resonance imaging.

The ‘total' column describes total number of scans available, including those from the pilot period and follow-ups, and those excluded for quality control. The ‘usable' column describes number of data files from the final protocol that survived quality control (except for those marked with an asterisk, which have not yet been subjected to quality control).

**Table 2:** Significant phenome-wide associations.

X class	X variable	Y class	Y variable	*r*	*t*	*N*	*P*(BF)
behav	Alcohol intake (previous evening)	fullmetab	Butane-2,3-diol	0.792	7.38	40	0.0000
bwcorr	Visual-2-visual-1	behav	Fatigue (after scan)	0.400	5.10	74	0.0129
bwcorr	Visual-2-dorsal attention	behav	Fatigue (after scan)	0.496	5.06	74	0.0157
bwcorr	Visual-1-cingulo-opercular	behav	Fatigue (after scan)	0.621	6.81	74	0.0000
bwcorr	Somatomotor-medial parietal	behav	TuesThurs	0.532	5.34	72	0.0036
food	Cashews	fullmetab	Glutamine	0.553	5.29	39	0.0048
food	Eggs	fullmetab	*N*-methylalanine	0.428	5.07	39	0.0152
food	Bacon	metab	C15: no enrichment	0.616	5.80	39	0.0002
food	Olives	wgcna	ME16: no enrichment	0.464	5.88	39	0.0002
fullmetab	Glutamic acid	bwcorr	DMN-visual-1	0.574	4.94	39	0.0293
fullmetab	Beta-alanine	bwcorr	Visual-2-fronto-parietal	0.527	5.17	39	0.0092
fullmetab	Aminomalonate	bwcorr	DMN-somatomotor	0.496	5.27	39	0.0052
immport	Antimicrobials	bwcorr	Salience-cingulo-opercular	0.452	4.84	39	0.0491
metab	C13:retinol biosynthesis	bwcorr	Visual-2-dorsal attention	0.505	4.84	39	0.0497
wgcna	ME45:no enrichment	bwcorr	Visual-2-parieto-occipital	0.353	4.84	39	0.0499
wgcna	ME52:protein catabolic process	bwcorr	Fronto-parietal-somatomotor	0.385	5.13	39	0.0113
wgcna	ME60:no enrichment	bwcorr	Fronto-parietal-somatomotor	0.473	5.83	39	0.0002
wgcna	ME28:no enrichment	fullmetab	Uric acid	0.502	4.93	48	0.0308
wincorr	Visual-2	wincorr	Somatomotor	0.553	5.98	84	0.0001
wincorr	Visual-1	wincorr	Somatomotor	0.478	5.22	84	0.0067
wincorr	Somatomotor	wincorr	Visual-1	0.478	4.88	84	0.0414

*r*, Pearson's correlation between variables; *t*, *t*-statistic from time-series regression; *N*, number of observations entering analysis; *P* (BF), Bonferroni-corrected *P* value. A listing of the strongest associations between measures across the entire data set, sorted by the X variable class. All tests listed here were significant after the Bonferroni correction for all 38,363 tests. Variable classes are abbreviated as: wincor, within-network connectivity; bwcorr, between-network connectivity; netdat, graph–theoretic measures on brain connectivity; gene, gene expression modules; metab, metabolite modules; fullmetab, individual metabolites; behav, behavioural/self-report measures.
