# Supplementary material for: Long-term neural and physiological phenotyping of a single human
Source: Nat Commun. 2015 Dec 9;6:8885. doi: 10.1038/ncomms9885 (PMC4682164; doi:10.1038/ncomms9885)
Supplement: Supplementary Information — Supplementary Tables 1-2 and Supplementary Note 1 [file ncomms9885-s1.pdf]

Supplementary Table 1: Modules identified using WGCNA analysis, with annotation using DAVID for pathways (including Reactome, Panther, KEGG, and BioCarta) and Gene Ontology (GO) terms, and hub genes for each network. P values are FDR-corrected. FE: Fold enrichment value computed using DAVID.

Mod #	N genes	Pathway	P (FDR)	FE	GO term	P (FDR)	FE	Hub genes
1	2025	HIV Infection (Reactome:6185)	0.009	1.8	transcription (GO:0006350)	0	1.5	TRRAP
		Processing of Capped Intron-Containing Pre-mRNA (Reactome:125)	0.01	2.1	regulation of transcription (GO:0045449)	0	1.4	SEC16A
		Regulation of activated PAK-2p34 by proteasome mediated degradation (Reactome:13635)	0.01	3.1	chromatin modification (GO:0016568)	0	2.1	CREBBP
		Ubiquitin proteasome pathway (P00060)	0.011	2.5	regulation of RNA metabolic process (GO:0051252)	0.001	1.3	IGF1R
		Gene Expression (Reactome:71)	0.012	1.6	regulation of transcription, DNA-dependent (GO:0006355)	0.002	1.3	ARHGAP35
		Signaling by Wnt (Reactome:11045)	0.013	2.5	positive regulation of transcription, DNA-dependent (GO:0045893)	0.002	1.6	
		APC/C (Reactome:9035)	0.013	2.5	positive regulation of gene expression (GO:0010628)	0.002	1.6	
		Cdc20 (Reactome:6850)	0.025	2.3	macromolecule catabolic process (GO:0009057)	0.002	1.5	
2	1364	Parkinson disease (P00049)	0.039	2.7	establishment of protein localization (GO:0045184)	0.001	1.7	ARL5A
		Cell Cycle, Mitotic (Reactome:152)	0.054	1.7	protein transport (GO:0015031)	0.001	1.7	COPB1
		Cdc20 (Reactome:6850)	0.067	2.7	protein catabolic process (GO:0030163)	0.016	1.6	VBP1
		Signaling by Wnt (Reactome:11045)	0.071	2.7	translation (GO:0006412)	0.017	1.9	STK17A
		DNA Repair (Reactome:216)	0.085	2.1	modification-dependent protein catabolic process	0.018	1.7	NDUFB5

					(GO:0019941)			
		Ubiquitin mediated proteolysis (KEGG:hsa04120)	0.093	2.3	modification-dependent macromolecule catabolic process (GO:0043632)	0.018	1.7	
		Oxidative phosphorylation (KEGG:hsa00190)	0.096	2.4	protein localization (GO:0008104)	0.019	1.5	
					cellular macromolecule catabolic process (GO:0044265)	0.024	1.6	
3	1256	no pathways enriched			lipid biosynthetic process (GO:0008610)	0.013	2.2	MAPK8IP3
								MICAL1
								SPPL2B
								SNAPC4
								UBXN11
4	1238	Spliceosome (KEGG:hsa03040)	0	3.5	RNA processing (GO:0006396)	0	2.3	EIF2S2
		Gene Expression (Reactome:71)	0.002	1.8	ribonucleoprotein complex biogenesis (GO:0022613)	0	3.4	TAF12
		HIV Infection (Reactome:6185)	0.002	2.2	mRNA metabolic process (GO:0016071)	0	2.3	TTC1
		Metabolism of non-coding RNA (Reactome:11052)	0.003	5.6	mRNA processing (GO:0006397)	0	2.4	TBCA
		Diabetes pathways (Reactome:15380)	0.017	1.7	RNA splicing (GO:0008380)	0	2.5	PPID
		Huntington's disease (KEGG:hsa05016)	0.024	2.2	ribosome biogenesis (GO:0042254)	0	3.5	
		Processing of Capped Intron-Containing Pre-mRNA (Reactome:125)	0.026	2.2	ncRNA metabolic process (GO:0034660)	0	2.7	
		Cell Cycle, Mitotic (Reactome:152)	0.035	1.6	protein folding (GO:0006457)	0	2.9	

5	1128	Hemostasis (Reactome:604)	0	2.8	coagulation (GO:0050817)	0.001	3.8	PRKG1
		Gap junction (KEGG:hsa04540)	0	3.7	blood coagulation (GO:0007596)	0.001	3.8	IGF2BP3
		Vascular smooth muscle contraction (KEGG:hsa04270)	0.01	2.8	hemostasis (GO:0007599)	0.002	3.8	PROS1
		Endocytosis (KEGG:hsa04144)	0.037	2.1	enzyme linked receptor protein signaling pathway (GO:0007167)	0.002	2.2	SLFN14
		Blood coagulation (P00011)	0.073	3.3	response to hormone stimulus (GO:0009725)	0.002	2.2	ABCC4
		Focal adhesion (KEGG:hsa04510)	0.091	2	protein amino acid phosphorylation (GO:0006468)	0.003	1.8	
					wound healing (GO:0042060)	0.003	2.7	
					intracellular signaling cascade (GO:0007242)	0.003	1.6	
6	923	no pathways enriched			RNA processing (GO:0006396)	0.001	2.2	ACADVL
					RNA splicing (GO:0008380)	0.006	2.6	DHX38
					mRNA processing (GO:0006397)	0.074	2.2	MIR3916
					mRNA metabolic process (GO:0016071)	0.09	2.1	TPM3P9
								SLC25A45
7	810	no pathways enriched			transcription (GO:0006350)	0	1.6	RICTOR
					regulation of transcription (GO:0045449)	0	1.5	TRIM33

			protein catabolic process (GO:0030163)	0.006	2	UBR2
			regulation of RNA metabolic process (GO:0051252)	0.008	1.5	DMXL1
			modification-dependent protein catabolic process (GO:0019941)	0.017	1.9	ZNF121
			modification-dependent macromolecule catabolic process (GO:0043632)	0.017	1.9	
			regulation of transcription, DNA-dependent (GO:0006355)	0.019	1.5	
			cellular protein catabolic process (GO:0044257)	0.02	1.9	
8	623	no pathways enriched	no GO terms enriched			HNRNPH1<br>PDXDC2P<br>ENTPD4<br>ARMCX4<br>SREK1
9	586	no pathways enriched	no GO terms enriched			LMAN2<br>NDUFB7<br>GSTP1<br>AAMP<br>TESC
10	485	no pathways enriched	positive regulation of cell death (GO:0010942)	0.032	2.5	IFNAR1
			positive regulation of programmed cell death (GO:0043068)	0.039	2.5	EVI2B
			cellular response to extracellular stimulus (GO:0031668)	0.041	6.1	ZNF217

					response to organic substance (GO:0010033)	0.042	2.2	KCTD12
					positive regulation of apoptosis (GO:0043065)	0.053	2.5	NBR1
11	433	Valine, leucine and isoleucine degradation (KEGG:hsa00280)	0.007	7.8	deoxyribonucleotide catabolic process (GO:0009264)	0.018	18.5	RNH1
								CYBA
								MBD3
								SSBP4
								SIPA1
12	425	mTOR signaling pathway (KEGG:hsa04150)	0.014	7	no GO terms enriched			INTS1
		Transcription (Reactome:1788)	0.026	4.1				MYBBP1A
								ULK1
								CLCN7
								PKD1
13	405	Taste transduction (KEGG:hsa04742)	0.001	9.9	sensory perception of taste (GO:0050909)	0	13.2	TTN
								LOC100132247
								PKD2L2
								TENM1
								MYH10

14	364	Signaling in Immune system (Reactome:6900)	0.006	2.8	immune response (GO:0006955)	0	2.7	ATP6V0D1
		Natural killer cell mediated cytotoxicity (KEGG:hsa04650)	0.069	3.3	defense response (GO:0006952)	0.009	2.4	TMEM176B
		Leukocyte transendothelial migration (KEGG:hsa04670)	0.075	3.7				IL17RA
		Pathways in cancer (KEGG:hsa05200)	0.078	2.3				TMEM176A
		Axon guidance (KEGG:hsa04360)	0.093	3.4				NUP62
15	187	no pathways enriched			no GO terms enriched			ZDHHC17
								DNAJB14
								DHX15
								FAM76B
								MIOS
16	166	no pathways enriched			no GO terms enriched			ERGIC1
								CFL1
								PIGS
								PPP1R11
								NMT1
17	133	no pathways enriched			no GO terms enriched			CPVL
								HNMT
								CD86
								ACSL4
								NLRC4
18	130	Integrin signalling pathway (P00034)	0.068	4	no GO terms enriched			ACTN1

								MPL
								PEAR1
								CMIP
								ANKRD33B
19	127	no pathways enriched			transcription (GO:0006350)	0	3.3	SMARCAD1
					regulation of transcription (GO:0045449)	0	2.9	ZNF441
					regulation of RNA metabolic process (GO:0051252)	0	3.4	ZBTB41
					regulation of transcription, DNA-dependent (GO:0006355)	0	3.4	GOPC
								ZNF33B
20	104	no pathways enriched			no GO terms enriched			TRIQK
								ITFG1
								HIPK3
								BNIP3L
								RMND5A
21	101	Cell Cycle, Mitotic (Reactome:152)	0	6.1	no GO terms enriched			CNTRL
								TPR
								AKAP9
								PCM1
								PHF3
22	85	no pathways enriched			chromosome organization (GO:0051276)	0	6.9	SMARCC2

			chromatin organization (GO:0006325)	0.001	6.9	YLPM1
			chromatin modification (GO:0016568)	0.016	6.9	RBBP6
			mRNA metabolic process (GO:0016071)	0.073	5.1	BRPF3
			RNA splicing (GO:0008380)	0.081	5.8	MAP4K4
23	72	no pathways enriched	no GO terms enriched			SNORA50<br>SCARNA3<br>SNORA64<br>SNORA20<br>SNORA18
24	56	no pathways enriched	no GO terms enriched			NCOA1<br>NCOR1<br>BPTF<br>ANKRD17<br>TAB2
25	54	no pathways enriched	no GO terms enriched			LYST<br>DMXL2<br>LRRK2<br>CREB5<br>PTPRE
26	48	no pathways enriched	no GO terms enriched			EFCAB14<br>ZBTB2<br>ATMIN<br>SSR1<br>ATF6
27	35	no pathways	response to virus	0.001	27.6	IFIT3

		enriched			(GO:0009615)			IFIT1<br>IFIT2<br>LAP3<br>RSAD2
28	30	no pathways enriched			no GO terms enriched			SKAP2<br>TLR5<br>RALB<br>DSE<br>CD36
29	28	Telomere Maintenance (Reactome:7970)	0	48.5	nucleosome organization (GO:0034728)	0.003	36.4	HIST1H4H
		Systemic lupus erythematosus (KEGG:hsa05322)	0	41.1	protein-DNA complex assembly (GO:0065004)	0.003	37.2	HIST1H3D
					DNA packaging (GO:0006323)	0.004	28.9	MEMO1
					chromatin assembly (GO:0031497)	0.004	38.9	HIST1H4A
					chromatin assembly or disassembly (GO:0006333)	0.005	26.6	C6orf48
					chromatin organization (GO:0006325)	0.007	11.2	
					nucleosome assembly (GO:0006334)	0.008	40.3	
					chromosome organization (GO:0051276)	0.016	8.7	
30	27	IL 5 Signaling Pathway (h_il5Pathway)	0.008	71.8	no GO terms enriched			CLC
		Asthma (KEGG:hsa05310)	0.084	37.6				CCR3<br>IL5RA<br>MS4A3<br>MS4A2

31	26	no pathways enriched			no GO terms enriched			PION<br>TMEM181<br>ATP8A1<br>GOLT1B<br>ANKAR
32	25	no pathways enriched			no GO terms enriched			CSF3R<br>PRAM1<br>INPPL1<br>RUSC2<br>ADAMTSL4
33	21	Natural killer cell mediated cytotoxicity (KEGG:hsa04650)	0	19.1	natural killer cell activation (GO:0030101)	0.071	113.7	PDGFRB
		Graft-versus-host disease (KEGG:hsa05332)	0.001	43.5				FCRL6
		Antigen processing and presentation (KEGG:hsa04612)	0.006	20.4				YPEL1
		Signaling in Immune system (Reactome:6900)	0.015	7.9				KIR3DL1
								KIR2DL1
34	19	Processing of Capped Intron-Containing Pre-mRNA (Reactome:125)	0.001	24.7	RNA splicing (GO:0008380)	0	22.2	NCL
		Influenza Infection (Reactome:6167)	0.001	17.3	mRNA processing (GO:0006397)	0	19.7	HNRNPM
		Gene Expression (Reactome:71)	0.005	7.8	mRNA metabolic process (GO:0016071)	0	17.1	HNRNPA0
		Spliceosome (KEGG:hsa03040)	0.058	17.3	RNA processing (GO:0006396)	0	11.5	STRAP

					nuclear mRNA splicing, via spliceosome (GO:0000398)	0.008	23.6	PDCD4
					RNA splicing, via transesterification reactions (GO:0000375)	0.008	23.6	
					RNA splicing, via transesterification reactions with bulged adenosine as nucleophile (GO:0000377)	0.008	23.6	
35	18	Signaling in Immune system (Reactome:6900)	0.043	11.9	response to bacterium (GO:0009617)	0	35	LTF
		Hemostasis (Reactome:604)	0.069	14.5	defense response to bacterium (GO:0042742)	0	52.8	OLFM4
					defense response (GO:0006952)	0	13.7	CRISP3
					immune response (GO:0006955)	0	9.8	CEACAM8
					aminoglycan catabolic process (GO:0006026)	0.006	120.8	BPI
					polysaccharide catabolic process (GO:0000272)	0.008	90.6	
					aminoglycan metabolic process (GO:0006022)	0.039	39	
					polysaccharide metabolic process (GO:0005976)	0.084	22.9	
36	16	no pathways enriched			no GO terms enriched			SNORA74A
								SNORA45
								SNORA53
								SNORA77
								SCARNA11

37	13	Signaling in Immune system (Reactome:6900)	0.084	11.9	no GO terms enriched	KLRD1<br><br>SH2D1B<br>CCL4<br>PRSS23<br>NADKD1
38	12	no pathways enriched			no GO terms enriched	CD33<br><br>FKBP15<br>OSCAR<br>GAB2<br>ARRB2

Supplementary Table 2. Clusters of compounds identified using affinity propagation clustering on metabolomics data, with pathway enrichment identiifed using IMPALA.

Cluster #	Compounds	Pathways	FDR
1	tocopherol gamma-, tocopherol alpha-, threitol, shikimic acid, pyrophosphate, malic acid, isothreonic acid, indole-3-acetate, fumaric acid, cholesterol, 2-ketoisocaproic acid	no enrichment	
2	trans-4-hydroxyproline, threonine, serine, oxoproline, myo-inositol, hexitol, glycine, citric acid, aminomalonate, alanine	Glycerophospholipid Biosynthetic Pathway (Wikipathways)	0
		Glycerophospholipid Biosynthetic Pathway (Wikipathways)	0
		gamma-glutamyl cycle (HumanCyc)	0
3	parabanic acid, oxalic acid, nicotinic acid, glycolic acid, alloxanoic acid, 2,4-diaminobutyric acid, 2,3-dihydroxypyridine	no enrichment	
4	sucrose, ornithine, lysine, histidine	ABC transporters - Homo sapiens (KEGG)	0.001
		Glucose Homeostasis (Wikipathways)	0.001
		Amino acid transport across the plasma membrane (Reactome)	0.003
5	palmitoleic acid, oleic acid, linoleic acid, capric acid, arachidonic acid	triacylglycerol degradation (HumanCyc)	0
		Sphingomyelin metabolism/ ceramide salvage (HumanCyc)	0
		phospholipases (HumanCyc)	0
6	urea, phosphate, methionine sulfoxide, hexuronic acid	no enrichment	

7	tyrosine, mannose, lactic acid, glucose, 1,5-anhydroglucitol	no enrichment	
8	valine, pseudo uridine, N-methylalanine, N-acetylornithine, methionine, leucine, isoleucine, indole-3-lactate, hippuric acid, glyceric acid, glutamic acid, 2-hydroxyvaleric acid	Endosomal/Vacuolar pathway (Reactome)	0
		Proton/oligonucleotide cotransporters (Reactome)	0
		Aminoacyl-tRNA biosynthesis - Homo sapiens (KEGG)	0
9	stearic acid, palmitic acid, myristic acid, isoheptadecanoic acid, heptadecanoic acid, beta-alanine	Transport of fatty acids (Reactome)	0
		Fatty acid biosynthesis - Homo sapiens (KEGG)	0
		Free fatty acid receptors (Reactome)	0.001
10	phenylalanine, lyxitol, hexonic acid, glycerol, ethanolamine, erythritol, butane-2,3-diol, 2-deoxytetronic acid, 2-deoxyerythritol	Glycerophospholipid Biosynthetic Pathway (Wikipathways)	0.018
		Glycerophospholipid Biosynthetic Pathway (Wikipathways)	0.034
		Glycerophospholipid Biosynthetic Pathway (Wikipathways)	0.095
11	tryptophan, taurine, proline, phenylethylamine, glutamine, creatinine, asparagine	Na+/Cl- dependent neurotransmitter transporters (Reactome)	0
		Transport of glucose and other sugars, bile salts and organic acids, metal ions and amine compounds (Reactome)	0
		Amino acid transport across the plasma membrane (Reactome)	0

12	xylulose, xylose, uridine, pelargonic acid, p-cresol, N-acetylmannosamine, methanolphosphate, lauric acid, hydroxylamine, hydroxycarbamate, glycerol-alpha-phosphate, dehydroabietic acid, benzoic acid	no enrichment	
13	behenic acid, arachidic acid	retinol biosynthesis (HumanCyc)	0.064
		fatty acid activation (HumanCyc)	0.064
		acyl-CoA hydrolysis (HumanCyc)	0.064
14	uric acid, threonic acid , succinic acid, fructose, cystine, acetoacetate	no enrichment	
15	N-acetylglycine, isocitric acid, 3-hydroxybutyric acid, 3-hydroxybutanoic acid, 2-hydroxybutanoic acid	no enrichment	

Supplementary Note 1: Survey Questions

1.1 Morning survey:
Question: Sleep quality
Answers: 1. Extremely poor, 2. Very poor, 3. Poor, 4. Average, 5. Good, 6. Very good, 7. Extremely good
Question: Soreness
Answers: 1. Extremely sore, 2. Very sore, 3. Sore, 4. Average, 5. Good, 6. Very good, 7. Extremely good

Question: Blood pressure
Answers: (free text)

Question: Pulse
Answers: (free text)
1.2 PANAS-X (administered after each scan)
For all questions, answers were:
1. Very slightly or not at all, 2. A little, 3. Moderately, 4. Quite a bit, 5. Extremely
afraid, scared, nervous, jittery, irritable, hostile, guilty, ashamed, upset, distressed, active, alert, attentive, determined, enthusiastic, excited, inspired, interested, proud, strong, cheerful, disgusted, attentive, bashful, sluggish, daring, surprised, strong, scornful, relaxed, irritable, delighted, inspired, fearless, disgusted with self, sad, calm, afraid, tired, amazed, shaky, happy, timid, alone, alert, upset, angry, bold, blue, shy, active, guilty, joyful, nervous, lonely, sleepy, excited, hostile, proud, jittery, lively, ashamed, at ease, scared, drowsy, angry at self, enthusiastic, downhearted, sheepish, distressed, blameworthy, determined, frightened, astonished, interested, loathing, confident, energetic, concentrating, dissatisfied with self
1.3 Post-scan survey
Question: Anxiety during scan
Answers: 1. Extremely anxious, 2. Very anxious, 3. Anxious, 4. Average, 5. Good, 6. Very good, 7. Extremely good

Question: Blood pressure
Answers: (free text)

Question: Thoughts during scan
Answers: (free text)
1.4 Evening survey
Question: Time spent outdoors (hours)
Answers: (free text)

Question: Psoriasis severity
Answers: 1. Extremely bad, 2. Very bad, 3. Bad, 4. Average, 5. Good, 6. Very good, 7. Extremely good

Question: Stress
Answers: 1. Extremely bad, 2. Very bad, 3. Bad, 4. Average, 5. Good, 6. Very good, 7. Extremely good

Question: Gut health
Answers: 1. Extremely bad, 2. Very bad, 3. Bad, 4. Average, 5. Good, 6. Very good, 7. Extremely good

Question: End of day report
Answers: (free text)

Question: Exercise (describe, leave blank for none)
Answers: (free text)

Question: Alcohol (standard drinks consumed)
Answers: (free text)

Question: Food (describe all ingredients eaten)
Answers: (free text)
